# Definitions, terminology, and related concepts of “racial health equity”: a scoping review protocol

**DOI:** 10.1186/s13643-023-02357-4

**Published:** 2023-09-30

**Authors:** Patricia C. Heyn, Elizabeth A. Terhune, Mahederemariam Bayleyegn Dagne, Christi Piper, Vivian A. Welch, Damian Francis, Ana B. Pizarro, Anita Rizvi, Nila Sathe, Omar Dewidar, Colleen Ovelman, Tiffany Duque, Tamara A. Baker, Robert W. Turner, Meera Viswanathan, Dru Riddle

**Affiliations:** 1 Center for Optimal Aging, Marymount University, 2807 N. Glebe Road, Arlington, VA 22207 USA; 2 Department of Physical Medicine and Rehabilitation, University of Colorado Anschutz Medical Campus, Aurora, CO USA; 3 Department of Orthopedics, University of Colorado Anschutz Medical Campus, Aurora, CO USA; 4 Strauss Health Sciences Library, University of Colorado Anschutz Medical Campus, Aurora, CO USA; 5 School of Epidemiology and Public Health, University of Ottawa, Ottawa, ON Canada; 6 Georgia College and State University, Milledgeville, GA USA; 7 Clinical Research Center, Fundación Valle del Lili, Cali, Colombia; 8 School of Psychology, Faculty of Social Sciences, University of Ottawa, Ottawa, ON Canada; 9 RTI International, RTI-UNC US Cochrane Affiliate, Chapel Hill, NC USA; 10 Bruyère Research Institute, Ottawa, ON Canada; 11 Cochrane, Central Executive Team, London, UK; 12 Department of Psychiatry, University of North Carolina, Chapel Hill, NC USA; 13 Department of Clinical Research and Leadership, George Washington University, Washington, DC USA; 14 Center for Translational Research, Texas Christian University, Fort Worth, TX USA

**Keywords:** Racial health equity, Health equity, Health justice, Scoping review, Landscape review, Racism, Discrimination, Health disparities, Definitions, Terminology

## Abstract

**Background:**

In the USA, access to quality healthcare varies greatly across racial and ethnic groups, resulting in significant health disparities. A new term, “racial health equity” (RHE), is increasingly reported in the medical literature, but there is currently no consensus definition of the term. Additionally, related terms such as “health disparities,” “health inequities,” and “equality” have been inconsistently used when defining RHE.

**Methods:**

The primary purpose of this scoping review is to investigate the current use and underlying concepts used to define racial health equity. The study will address two key questions: (1) “What terminology and definitions have been used to characterize RHE?” and (2) “What knowledge gaps and challenges are present in the current state of RHE research and theory?” The review will collect and analyze data from three sources: (1) websites from key national and international health organizations, (2) theoretical and narrative published articles, and (3) evidence synthesis studies addressing interventions targeting racial health equity and minority stakeholder engagement.

**Discussion:**

Defining “racial health equity” and related terminology is the first step to advancing racial health equity within the USA. This review aims to offer an improved understanding of RHE constructs and definitions, bringing greater unity to national racial health equity research efforts across disciplines.

**Systematic review registration:**

This protocol is registered with the Open Science Framework at https://osf.io/7pvzq.

**Supplementary Information:**

The online version contains supplementary material available at 10.1186/s13643-023-02357-4.

## Background

The concept of racial health equity (RHE) within the USA emerged in response to persistent disparities in health outcomes along racial lines. By 2003, the Institute of Medicine’s “unequal treatment” [1] drew attention to the fact that individuals of color consistently experienced worse health outcomes and received lower-quality care than their white counterparts. The SARS-COVID-19 pandemic further exacerbated inequities along racial lines [2, 3], and global racial justice protests further drew attention to RHE as a research field and call to action. Specifically, RHE is a component of health equity, a variable concept that focuses on eliminating unfair disparities in health based on racial, environmental, socioeconomic, or structural factors beyond an individual’s control.

**Fig. 1:**
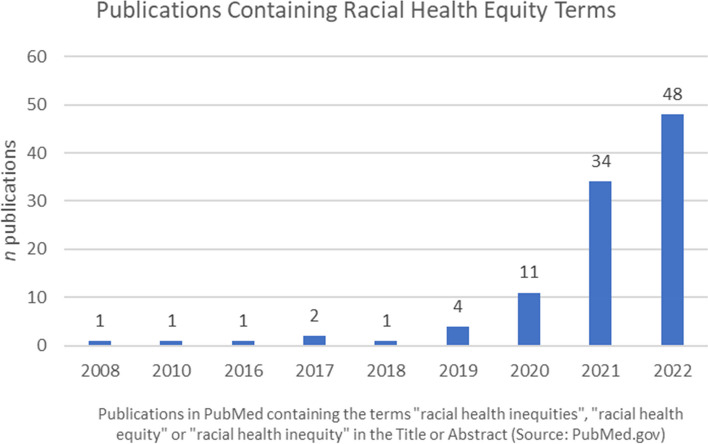
Publications containing racial health equity (RHE) terms have greatly increased in recent years. Results include all publications within PubMed containing indicated terms within the title or abstract. Source: PubMed.gov. Data retrieved January 27, 2023

In 2021, the White House released Executive Order #13,985 to advance health equity and to provide government support for people of color and others who have been historically underserved, marginalized, or affected by persistent poverty and inequality [4]. This order was extended in 2023 to establish equity-focused leadership plans within government agencies, creating economic rural opportunities and equity-focused urban developments, advancing civil rights, and promoting equity in data [5]. In the wake of these executive orders, government and private organizations, including the Robert Wood Johnson Foundation [6], and the United States Department of Health and Human Services [7, 8], have launched initiatives addressing racism and health inequities. Research studies specifically addressing racial health equity have also increased exponentially, while there were 0–2 articles per year containing RHE terms between the years 2008–2018, by 2022 there were 48 articles containing these terms (Fig. 1).

The effects of racism and discrimination are believed to be one of several social determinants of health (SDOH), which are non-medical factors that influence health outcomes. SDOH encompasses the environmental conditions in which people are born, work, live, and age [9]; 80–90% of a person’s health and well-being is determined by these factors [10]. There are several conceptual models to explain key SDOH that are believed to underlie health inequalities and explain how racism, a social construct not influenced by biology, impacts health equity. These social determinants include structural determinants (e.g., governmental policies, housing availability, socioeconomic status), intermediary determinants (e.g., material conditions, food availability), and systematic barriers that underlie root or upstream causes of health inequities [11]. Systemic or implicit racism negatively and unfairly affects the health of racialized groups in the USA and perpetuates racial health disparities [12]. The effects of racism are thus included as a SDOH, with effects ranging from unfair barriers to employment and education, as well as access to healthcare [13, 14].

Despite the growing attention on health equity and the emergence of research to study health disparities, there is not yet a shared definition of terms, concepts, or conceptual frameworks. This may be due in part to the multidisciplinary nature of health equity research, with studies originating from investigators from both clinical and non-clinical backgrounds (e.g., anthropology, public health, sociology, economics, epidemiology, and history). Additionally, three or more concepts underlie “racial health equity” (“racial,” “health,” and “equity”), which may be based on different constructs, values, or principles. For example, “health” can be further characterized into at least three domains, including physical, social, and mental well-being [15]. “Health equity” is also defined differently across health organizations and across research fields.

Interventions to mitigate health inequities vary across sectors, and the metrics of outcomes differ across disciplines. While many studies have been undertaken to reduce gaps in centering RHE, they may lack input and representation from other key disciplines outside of medicine, such as education and public health as well as from diverse stakeholders across hierarchy and settings whose input can inform the U.S. healthcare system for advancing racial health equity. A national consensus is lacking on what constitutes racial health equity. Clear and consistent descriptions of definitions, terms, constructs, and frameworks are needed to incorporate and integrate RHE concepts across organizations and disciplines to begin to establish a national consensus to center, guide, and advance RHE.

This protocol represents the first known effort to systematically catalog, evaluate, and map RHE terminology in its current and historical forms. We will use a multi-part search strategy to analyze the use of RHE terms within major health websites, theoretical articles, and evidence syntheses. Alignment and clarity of the current knowledge of RHE definitions and terms—with the understanding that these terms will continue to evolve over time—is a crucial first step to driving the development and implementation of relevant interventions for high-risk groups to achieve positive health outcomes.

### Aims and objectives

In this study, we propose to conduct a landscape analysis, which is an evidence synthesis method that identifies trends, gaps, and opportunities within a specified research field. Our analysis will collect definitions of “racial health equity” identified in key public health organization websites, original theoretical articles, narrative reviews, and recent evidence synthesis studies from medical and social science databases. Full inclusion and exclusion criteria are provided in Tables 1 and 2, and Supplemental Table 3.

**Table 1:** Inclusion and exclusion criteria for websites review (Aim 1)

	**Inclusion criteria**	**Exclusion criteria**
Website type	Well-established public health organizations (ex: CDC, WHO, NIH), organizations that guide and inform health care (ex: CMS), organizations with a focus on improving health and provide best practices (ex: Cochrane), non-profit health organizations (government, national, associations, professional societies)	For-profit health organizations (ex: pharmaceuticals), commercial websites, state health organizations, universities, hospitals
Setting	United states and organizations that guide US health care	Websites that are public but not in the USA or do not guide US health care
Date	No date restriction	N/A
Language	English	Other languages

**Table 2:** Inclusion/exclusion criteria for theoretical articles (SA 2)

	**Inclusion criteria**	**Exclusion criteria**
Article type	Primary research article Theoretical article Narrative reviews Editorials	Conference abstracts Video or other media Book chapters Dissertations Evidence syntheses
Content	Contains the definition of “racial health equity” or separate definitions of “health equity” within the context of race/ethnicity/minority communities Includes words of concept/definition/framework/terminology	No definition of “racial health equity”, “health equity,” or related terms Culture or acculturation focus The article focused on measuring outcomes without defining terms
Setting	No setting explicitly mentioned, or relevant to the USA (includes countries high on the human development index per WHO guidelines)	The article explicitly set within non-highly developed settings
Date	No date restriction	N/A
Language	English	Other languages
Accessibility	Full text available through the University of Colorado or Marymount University libraries	Full text unavailable through UC or MU libraries

Our RHE terminology study is part of a larger project, “Centering racial health equity in systematic reviews of interventions” conducted by the Cochrane US Network and funded by the Robert Wood Johnson Foundation which includes four scoping or landscape reviews including the present study. The remaining reviews focus on (1) stakeholder engagement [16], (2) logic models of RHE [17], and (3) healthcare interventions to promote equity in racialized populations [18]. To our knowledge, our terminology and definition study is the first to evaluate RHE constructs through systematic reviews and a landscape analysis. Our overarching goal is to determine how RHE constructs are used, defined, determined, and applied in the current state of knowledge by addressing the following key questions (KQs):

- KQ1: What terminology and definitions have been used to characterize racial health equity in the following:
  - Public guidance documents, reports, and information content in websites produced by key public health organizations (e.g., government, private, non-profit) involved in guiding public health, medicine, and evidence-based practices in the USA
  - Theoretical or conceptual original publications
  - Evidence syntheses on interventions focused on addressing racial health equity?
- KQ2: What knowledge gaps and challenges are present in the current state of RHE research, practice, and theory?

To answer these questions, we propose the following Specific Aims (SA):

- SA 1: To identify and summarize RHE terminology used by key health organizations involved in guiding public health, medicine, and evidence-based practices
- SA 2: To identify and summarize RHE terminology and definitions from primary original, theoretical/conceptual articles
- SA 3: To identify and summarize RHE terminology and definitions from evidence synthesis studies
- SA 4: Summarize findings from Aims 1–3 to identify gaps and challenges in the current literature and to make recommendations for future research.

#### Justification and rationale

The study of racial health equity (RHE) is a burgeoning area with diverse, disparate definitions of key terms. Establishing consensus definition(s) of racial health equity will benefit community members, researchers, and policy makers by (1) allowing for precise measurements of intervention success against the shared definition, (2) allowing for the goals of equity research and policy to align to the shared definition, and (3) increasing the clarity of racial health equity research and policy language. Each of these benefits has the potential to enhance the impact of health equity interventions on racially minoritized populations.

## Methods

### Search strategy and data extraction

#### Review of health websites (Aim 1)

A list of major health websites with relevance to public health in the USA will be compiled from a search engine (Google) using the following search terms: “public” + ”health” + ”organizations” + ”United States”. Inclusion and exclusion criteria for websites is provided in Table 1. Websites to be analyzed will be limited to not-for-profit (e.g., Robert Wood Johnson Foundation), government (e.g., National Institutes of Health, Centers for Disease Control), academies (e.g., American Academy of Pediatrics), or evidence synthesis (e.g., Cochrane) organizations. Global organizations with relevance to the USA (e.g., World Health Organization) will be included. Excluded websites will include corporate or for-profit organizations, state-level government websites, and hospital or university websites. Additional websites fitting the inclusion criteria will be added based on input from the study team. Categories and corresponding numbers of websites to be included in our analysis are provided in Supplemental Table 1.

Definitions for terms including “race”/”ethnicity,” “racism”/”discrimination,” and “racial health equity”/ “health equity” will be collected from each website, when present. Exact URLs, date of access, and any cited sources will be collected. The homepage, links to the different topics on the home page, and search functions will all be utilized to find definitions. Reports or links on the websites will also be searched for relevant definitions. Definitions will be reported as missing if they cannot be located after > 1 h of search time. All definitions will be collected in an Excel spreadsheet.

To assess the ease of locating RHE definitions on each website, we developed an ease-of-access website rating tool. Briefly, definition accessibility will be rated as “very easy,” “easy,” “medium,” “hard,” or “very hard” based on the location of the definition (homepage, external report, etc.), if present, and the time required to find the definition was not very long.

#### Theoretical articles (Aim 2)

SA 2 and 3 will include systematic database searches for relevant literature. For SA 2, we will perform a search of theoretical and narrative articles with no restrictions on the date of publication. Details are provided below:

##### Search strategy

MEDLINE (via Ovid MEDLINE® ALL, 1946 to present), Embase (via Embase.com, 1947 to present), Global Health (CABI), and PsycINFO (via Ovid, 1806 to present) will be used for the search strategy of primary articles and narrative reviews. The search will be developed and run by an experienced medical librarian. Subject headings and keywords will be used to search each database when available. The initial search strategy will be built in Ovid MEDLINE and then translated to additional databases. The MEDLINE search strategy for SA 2 is available in Supplemental Table 2.

##### Inclusion/exclusion criteria

We will include theoretical and research articles in our definitions search under SA 2. This will include theoretical articles, primary research articles, narrative reviews, and editorials. Abstracts, dissertations, books, and other media will be excluded. Articles will be included if they have any definitions of “racial health equity” or “health equity” in the context of racial or ethnic health. We will not exclude articles based on date of publication or setting, unless the setting is explicitly mentioned as pertinent to non-highly developed settings. Searches will be limited to English language results. We will also perform hand searches for relevant gray literature under SA 2. Searches will be conducted via Google Scholar and will include additional narrative reviews, editorials, and/or book chapters following the inclusion/exclusion criteria*.* Full inclusion/exclusion criteria for SA 2 are provided in Table 2.

We will use the Covidence systematic review platform [19] to compile and screen articles for abstract and full-text review under the supplemental search. Covidence is a web-based collaboration software platform that streamlines the production of systematic and other literature reviews. A Covidence license is available to the study team through the University of Colorado Strauss Health Sciences Library. Two reviewers per title will review the title/abstracts for study inclusion, followed by a full-text review by two reviewers for final study inclusion. Any disagreements and conflicts will be resolved by discussion and consensus agreement.

##### Data extraction

Key data extracted will include, but not be limited to author and journal information (e.g., article title, first and last author, countries of authors, areas of expertise, field of expertise, journal, journal field), article information (e.g., purpose of review, focus of review, scope of review, key questions of review, article conclusions), and definitions (e.g., “health equity,” “race/racial/ethnicity,” “racial health equity,” and appropriate citations and page numbers of definitions). If present, we will also collect definitions and citations for related RHE terms, such as racial health *justice* or -*disparities*. We will use a REDCap database [20, 21], hosted at the University of Colorado Denver, to extract key information from our included studies (SA 2 and 3). REDCap (Research Electronic Data Capture) is a secure, web-based software platform designed to support data capture for research studies, providing (1) an intuitive interface for validated data capture, (2) audit trails for tracking data manipulation and export procedures, (3) automated export procedures for seamless data downloads to common statistical packages, and (4) procedures for data integration and interoperability with external sources. Data extraction will first be tested for congruity between two independent reviewers for at least 10 articles. Data will be extracted by one reviewer and independently verified by a second reviewer.

#### Evidence synthesis studies (Aim 3)

For SA 3, we will analyze definitions of health equity found within evidence syntheses identified by our team’s overlapping studies of racial health equity interventions within evidence syntheses and methods guidance documents. Evidence syntheses identified under these studies will be reviewed and included for data extraction if definitions of RHE terminology are present.

##### Search strategy

Included evidence syntheses will be published in 2020 onward, while method guidance documents will have no date restriction. Title and abstract screening will be conducted in Distiller (DistillerSR. Version 2.35. DistillerSR Inc., 2023, to be accessed January–June 2023. https://www.distillersr.com/) in combination with simultaneous study searches of racial health equity interventions and methods guidance documents [16, 18]. These studies will include evidence syntheses of health interventions to promote health equity for racialized groups that were published since 2020. Multiple independent reviewers (two per title) will review titles and abstracts for potential inclusion, followed by a full-text review for a final determination of study inclusion. Any disagreements and conflicts will be resolved by discussion and consensus agreement. Three members of the study team will then review all articles included for definitions of racial health equity. We anticipate that many of these studies will include references to secondary studies for their definitions, and we will utilize our team research librarian for assistance in pulling these referenced articles.

##### Data extraction

Data will be extracted into an Excel and REDCap database, as in SA 2 (see above). Data extracted will include, but not be limited to author and journal information (e.g., article title, first and last author, countries of authors, areas of expertise, field of expertise, journal, journal field), syst evidence synthesis information (e.g. type of evidence synthesis, type of intervention, purpose of intervention, health condition targeted), definitions (e.g., “health equity,” “race/racial/ethnicity,” “racial health equity,” and appropriate citations and page numbers of definitions).

##### Statistical analysis

Statistical analysis of SA 1–3 will be limited to summary and frequency statistics of the selected data. GraphPad Prism software (San Diego, CA, USA, www.graphpad.com) will be used to generate summary statistics and generate figures, when appropriate.

##### Quality assessment and risk-of-bias analysis

Due to the nature of the articles we will be reviewing, we will not be conducting quality assessments or risk-of-bias analyses. At this time, we are not aware of any quality or risk-of-bias assessment tools designed for qualitative, theoretical articles that would report terminology information.

##### Thematic analysis

Extracted definitions will be analyzed for recurring words using thematic analysis software. Word cloud software will also be used to visualize frequently used words or concepts.

##### Stakeholder involvement

We recruited diverse leaders in global health equity to our team as regards cultural and linguistic background, country, area of expertise, and gender. Additionally, we recruited a talented advisory board with diverse areas of expertise in health equity. This advisory board helped ensure that research efforts were not duplicated and that relevant data was collected. We followed an inclusive process to design this protocol and incorporate feedback.

## Discussion

Determining the current understanding of “racial health equity” is a first step towards promoting actionable and measurable goals to reduce health disparities within the USA. We anticipate that many websites will cite RHE terms but not provide explicit definitions. We also anticipate that most theoretical and systematic review articles that include definitions will cite a secondary source. Collecting data from these secondary sources will be necessary for mapping definitions and identifying shared sources and concepts.

Our study has several limitations due to the nature of the definitions and linguistics, which are variable by field and constantly evolving. Our website search (SA 1) will be limited to major health organizations that appear in our search or are known to the study team, which will be influenced by our areas of expertise and biases. Our search will also be limited to one accession timepoint, and we acknowledge that definitions may be updated at any time. We will be transparent about this fact by collecting accession date information for all definitions. We will only review websites and articles in English; additionally, we will only collect evidence synthesis articles that have been published since 2020. We acknowledge that relevant articles may be missed, but we anticipate that we will be able to collect the most relevant definitions given the recent expansion of RHE terms in the literature (see Fig. 1).

### Supplementary Information

**Additional file 1: Table S1.** List of websites for review (SA 1). **Table S2.** Keywords for theoretical article search (SA 2). **Table S3.** Inclusion/exclusion criteria for SA 3 according to PICOT guidelines. Inclusion criteria for systematic reviews is provided from parent study (*Centering Racial Health Equity in Systematic Reviews) *[14].

## Data Availability

The data used for this study will be extracted from publications within the search databases as outlined in the “Methods” section. Extracted data will be made available via a University of Colorado library server.
