# Supplementary material for: Definitions, terminology, and related concepts of “racial health equity”: a scoping review protocol
Source: Syst Rev. 2023 Sep 30;12:185. doi: 10.1186/s13643-023-02357-4 (PMC10542690; doi:10.1186/s13643-023-02357-4)
Supplement: Supplementary file 1 — Additional file 1: Table S1. List of websites for review (SA 1). Table S2. Keywords for theoretical article search (SA 2). Table S3. Inclusion/exclusion criteria for SA 3 according to PICOT guidelines. Inclusion criteria for systematic reviews is provided from parent study (Centering Racial Health Equity in Systematic Reviews) [14]. [file 13643_2023_2357_MOESM1_ESM.docx]

Supplemental File 1

**Supplemental Table 1:** List of websites for review (SA 1)

| Public health Organizations (Google, Expert recommendation, Commonly known health organizations | | |
| --- | --- | --- |
| Search date = March 9, 2023 – May 08, 2023 | | |
| 1 | Government Organizations | 40 |
| 2 | Non-profit | 23 |
| 3 | Private foundation | 2 |
| 4 | Not-for-profit | 4 |
| 5 | Other | 2 |

**Supplemental Table 2:** Keywords for theoretical article search (SA 2)

| MEDLINE (via Ovid MEDLINE® ALL, 1946 to March 1, 2023) | | |
| --- | --- | --- |
| Search date = March 2, 2023 | | |
| 1 | Racial Groups/ or exp "Race Relations"/ or "Race Factors"/ or "Health Disparate, Minority and Vulnerable Populations"/ or exp "Asian American Native Hawaiian and Pacific Islander"/ or "Black or African American"/ or exp "Hispanic or Latino"/ or exp "Indigenous Peoples"/ | 154223 |
| 2 | (racial* or race or racism or racist or minority or minorities or ethnic or ethnicit* or global).ti,ab. | 862469 |
| 3 | (Asian or "African American*" or Black or Blacks or Hispanic* or Latino* or Latinx or "Mexican American*" or indigenous or "American Indian*" or "Native American*" or "Alaska* Native*" or "Pacific Islander*" or Arab or Arabs or Jews or Jewish or "vulnerable population*").ti,ab. | 433936 |
| 4 | 1 or 2 or 3 | 1220636 |
| 5 | *"Health Equity"/ or *"Health Status Disparities"/ or *"Health Inequities"/ or *"Healthcare Disparities"/ | 27308 |
| 6 | (health adj4 (equity or inequit* or inequalit* or equalit* or disparit* or equities or equitable or discrimination or prejudice* or disadvantage*)).ti,ab. | 49984 |
| 7 | 5 or 6 | 66259 |
| 8 | *"Terminology as Topic"/ or *"Vocabulary"/ | 31558 |
| 9 | (terminolog* or etymolog* or nomenclature* or vocabular* or "concept map" or "concept maps" or conceptualization* or conceptualize* or taxonom* or ((conceptual or theoretical) adj3 (framework or model or report))).ti,ab. | 232777 |
| 10 | (define* or defining or definition*).ti. | 72214 |
| 11 | 8 or 9 or 10 | 320683 |
| 12 | 4 and 7 and 11 | 892 |
| 13 | 12 and English.lg. | 885 |
| 14 | remove duplicates from 13 |  |

**Supplemental Table 3:** Inclusion/exclusion criteria for SA 3 according to PICOT guidelines. Inclusion criteria for systematic reviews is provided from parent study (*Centering Racial Health Equity in Systematic Reviews).*[14]

| ***PICOT* category** | **Inclusion** | **Exclusion** |
| --- | --- | --- |
| **Population** | Evidence syntheses focused on racialized or minoritized populations  (i.e., populations that face discrimination or exclusion due to race/ethnicity) of any age AND interventions to address health inequities for these populations  Or  Primary literature, or evidence that report or imply universal approaches to conduct analysis to assess effects on racial health inequities/equity/equality*  Or  Methods documents/guidance that addresses considerations (design of logic models/frameworks for addressing racial health equity in evidence syntheses, stakeholder engagement in evidence syntheses, definitions of racial health equity, methods used to evaluate effects on racial health inequities in evidence syntheses) relevant for this project (i.e., addressing syntheses and racial health equity)^a^ | Evidence syntheses that describe race and ethnicity of the included studies with no methods to understand effects of interventions for racialized populations  Evidence syntheses that solely report race/ethnicity subgroup analyses and these subgroup analyses do not specifically address improving racial health inequities (e.g., subgroup analyses solely report outcomes by race/ethnicity)  Evidence syntheses that only report that more research/future research addressing race/ethnicity or health equity is needed    Guidance or methods documents that do not address racial health equity and methodologic areas of interest (definitions, frameworks, etc.) |
| **Intervention** | Study or review must address an aspect of health equity, including interventions; discussion of terms/concepts, policy or prevention  Interventions (individual, system-level, community level, policy level, preventive health services) addressing improving/mitigating health inequities for racialized/minoritized populations (including clinical, public health, social or structural inequities such as employment and education) | *Aim 3:* Not assessing effects of an intervention on racial health inequity    Evidence syntheses addressing exposure or association will not be included    Evidence syntheses that focus on individual-level strategies to address racism or unconscious bias such as cultural competence training |
| **Comparison** | **—** | **—** |
| **Outcomes** | Includes terms and conceptualizations of racial health equity  syntheses must report a health outcome (defined as changes (or lack of change) in current or future health status of individuals or groups of persons that are attributable to medical care or intervention. Health outcomes include elements such as physiologic or patient-reported measures; mortality and morbidity; physical, mental and social functioning; and quality of life.^b^), syntheses (e.g., scoping reviews) that report summary statements addressing direction of effect of health outcomes (e.g., 10 papers reported positive effects on blood pressure for Latinx participants) should be included    Methods-focused documents must address one of the following:   - Methods for engagement of stakeholders - Definitions of racial health equity/inequity - Frameworks and logic models addressing improving racial health equity/inequities | No definition of RHE terminology  No health outcome (i.e. effects on inequities in educational or employment opportunities or income will not be included)    Syntheses that only report cost outcomes  Papers that do not report relevant methods information (e.g., definitions/concepts, frameworks) |
| **Time** | Last 2 years for syntheses of effects of interventions to mitigate health inequities    No date limits for methods documents/guidance^c^ | Not published in 2020 or later (for evidence syntheses) |
| **Setting** | Relevant to the US health system ([highly developed countries](https://hdr.undp.org/data-center/human-development-index#_blank)—Human Development Index; this includes Switzerland, Norway, Iceland, Hong Kong, China (SAR), Australia, Denmark, Sweden, Ireland, Germany, Netherlands, Finland, Singapore, Belgium, New Zealand, Canada, Liechtenstein, Luxembourg, United Kingdom, Japan, Korea (Republic of), United States, Israel, Malta, Slovenia, Austria, United Arab Emirates, Spain, France, Cyprus, Italy, Estonia, Czechia, Greece, Poland, Bahrain, Lithuania, Saudi Arabia, Portugal, Latvia, Andorra, Croatia, Chile, Qatar, San Marino, Slovakia, Hungary, Argentina, Türkiye, Montenegro, Kuwait, Brunei Darussalam, Russian Federation, Romania, Oman, Bahamas, Kazakhstan, Trinidad and Tobago, Costa Rica, Uruguay, Belarus, Panama, Malaysia, Georgia, Mauritius, Serbia, Thailand) | Syntheses reporting research primarily conducted in non-highly developed countries |
| **Study Design** | Evidence syntheses (including systematic reviews, scoping reviews, rapid reviews, umbrella reviews, landscape reviews)^d^    Protocols for evidence syntheses will be included    Methods documents/guidance | Narrative reviews, environmental scans (although these will be searched for potentially eligible systematic reviews) |
| **Language** | English | Other languages |

^a^ Note that some syntheses or potential methods documents may imply, but not explicitly state, analysis of racial health equity/inequity considerations or the use of methods to address racial health equity/inequity; include these studies at the title/abstract level.

^b^ Definition adapted from definitions of health outcomes in Cochrane Methods Glossary (https://methods.cochrane.org/pro/sites/methods.cochrane.org.pro/files/uploads/Glossary.pdf)

^c^ Note that we will need to conduct supplemental searches for methods documents; each review team may choose to conduct specific supplemental searches for their topic areas
